# Evaporation of a Liquid Droplet

**DOI:** 10.6028/jres.081A.015

**Published:** 1977-04-01

**Authors:** Richard Kayser, Herbert S. Bennett

**Affiliations:** Institute for Materials Research, National Bureau of Standards, Washington, D.C. 20234

**Keywords:** Confluent hypergeometric functions, droplet, moving boundary problem, preheat, transient heat conduction

## Abstract

Two idealized models for the preheat stage of liquid droplets are analyzed theoretically. These models contain the effects of transient heat conduction and evaporation. It is assumed that the droplet surface area decreases linearly with time. This assumption necessitates the solution of moving boundary problems. These models, however, do not consider gas-phase mass transport. In the finite-gradient model, the temperatures of both the droplet and surrounding hot gases vary spatially and temporally. In the zero-gradient model the gas temperature varies spatially and temporally but the droplet temperature varies only temporally, i.e., the droplet temperature is spatially uniform. Numerical examples, which require extensive calculations of confluent hypergeometric functions, are presented for typical values of the droplet latent heat and evaporation rate constant. The temperature profiles given by the finite-gradient and zero-gradient models agree to within 20 percent of each other for all cases examined.

## 1. Introduction

The ignition of a droplet of conventional fuel consists essentially of two stages [1]^1^. During the first or preheat stage heat flows from the hot surrounding gas to the droplet, causing the droplet temperature to rise and liquid fuel to evaporate from the droplet surface. In the second stage, ignition occurs in the gaseous mixture of fuel and oxidizer surrounding the droplet. The preheat stage is dominated by transient processes and provides the motivation for the subject of this paper.

Wise and Ablow [2], Parks, et al. [3], and Waldman [4] have previously analyzed the effects of transient droplet heating by neglecting the existence of internal circulation and by neglecting selected terms in the heat conduction equations. In addition they assume that the droplet surface regresses linearly with time. In a previous publication, Bennett and Kayser [5] included the effects of internal circulation but also neglected evaporation and regression of the droplet surface. We now include these effects in the present paper.

The neglect of internal circulation can in many instances lead to gross underestimates of the heat transfer rates within the droplet. Available experimental evidence indicates that in most cases the heat transfer rate inside the droplet is much faster than is possible with heat conduction alone. El Wakil, et al. [6] observed vigorous circulation within the droplet and showed that the droplet temperature is uniform even during the initial transient period. This indicates that the rate at which heat is transferred to the droplet is much slower than the rate of internal mixing. As pointed out by Law [7], assuming the droplet temperature to be uniform in a sense circumvents the difficult task of describing internal circulation but still includes the effects due to its presence; that is, uniform droplet temperature represents the limit of rapid internal circulation.

Two models for the transient preheat stage are presented. In the first model, called the finite-gradient model, the effects of transient heat conduction and evaporation with linear surface regression rate are analyzed. The droplet and gas temperatures vary spatially and temporally and the effects of internal circulation are neglected. In the second model, called the zero-gradient model, we include the effects of transient heat flow, evaporation, and internal circulation. The latter is included by assuming the droplet temperature to be temporally varying but spatially uniform as discussed above. The gas temperature is again both time and space dependent.

The results of the finite-gradient and zero-gradient models are applied to the preheat stage of droplets in hot gases. The temperature profiles are used to predict the time duration between introduction of the droplet into a hot atmosphere and its reaching a given temperature. In addition we compare the analogous times predicted by a particularly simple and accurate approximation to the exact expression derived for the zero-gradient model.

In the next section we describe the physical assumptions for the preheat stage common to both models. Section 3 contains the mathematical description of these assumptions. We derive in section 4 the temperature profiles of the droplet and gas predicted by the finite-gradient model while in section 5 we give the corresponding expressions for the zero-gradient model. We present in the last section numerical examples for both models. The temperature profiles obtained are very similar for both models and are within 20 percent of each other in all cases examined.

## 2. Preheat Stage

Williams has examined the problem of the assumptions underlying droplet vaporization and combustion theories in some detail [8]. In models for preheating, many researchers consider the droplet to be a sphere with temperature independent thermal properties and neglect diffusion effects [1]. Others include thermal diffusion effects but later in their theoretical analyses neglect some terms in the diffusion equations [3]. In addition, most theoretical investigators neglect the influence of gravity, forced convection, and radiant heat transfer. Combustion chambers and experimental techniques such as freely falling droplets exist to eliminate gravitational effects. Proper selection of some experimental parameters such as ambient pressure and initial droplet size may minimize the other effects not included in the model for preheating. Several researchers have shown that except for the very heavy fuel oils, radiant heat transfer from the hot gas or from adjacent droplets is negligible [7, 9].

The models examined in this paper contain only thermal diffusion effects. We assume that a spherical liquid droplet of initial radius *r* = *r_d_*_0_ is inserted at time *t* = 0 into a hot atmosphere (e.g., air). The initial temperature of the droplet at time *t* = 0 is T*_d_*_0_. The hot gas is unbounded and initially at a constant temperature *T_g_*_0_. For *t* > 0 the temperature at infinity is kept at *T_g_*_0_.

The inclusion of evaporation demands that the droplet radius change with time. In the present work a linear surface regression rate is assumed:

$$R^{2}\left(t\right)=r_{d0}^{2}=k_{e}t$$ (1)

*R*(*t*) is a direct specification of the motion of the interface *r* = *R*(*t*) with time and *k_e_* denotes the evaporation rate constant. A self-contained theory would obtain *R*(*t*) indirectly by adding to the boundary conditions at the interface the requirement that the local vapor pressure be determined in the solution process. A moving boundary problem in which *R*(*t*) is directly specified falls into the general class of problems called inverse-Stefan problems while those in which it is determined in the solution process are called Stefan problems [10]. It must be stressed at this point that the solutions which we will obtain will be applicable only in those time regimes for which eq (1) is an accurate representation of the actual surface regression rate.

We denote the density, specific heat, and the thermal conductivity of the droplet respectively by *d_d_, C_d_*, and *K_d_* and of the gas respectively by *d_g_, C_g_* and *K_g_.* We assume that these thermal properties remain spatially and temporally constant during preheat. In addition, the ambient pressure of the host gas does not change. Using these and the above assumptions we seek to compute the dependence of the temperature *T*(*r, t*) upon the radial distance *r* and time *t.* In particular, we shall compute the time required for a droplet to reach a given temperature.

## 3. Theoretical Analysis

We first consider the finite-gradient model. The distribution of temperature within the system is governed by the Fourier heat conduction equation for the case in which the isothermal surfaces are concentric spheres. Let *T_d_*(*r*, *t*), *T_g_*(*r*, *t*), $a_{d}^{2}=\left(K_{d}/d_{d}C_{d}\right)$, and $a_{g}^{2}=\left(K_{g}/d_{g}C_{g}\right)$ denote the temperatures and thermal diffusivities of the droplet and surrounding gas respectively. The governing equation for *t* ≥ 0 and 0 ≤ *r* < *R* (inside the droplet) reads

$$\frac{\partial T_{d}\left(r,t\right)}{\partial t}=a_{d}^{2}\left(\frac{\partial^{2}T_{d}\left(r,t\right)}{\partial r^{2}}+\frac{2}{r}\frac{\partial T{\;}_{d}\left(r,t\right)}{\partial r}\right)$$ (2)

and for *t* ≥ 0 and *r* > *R* (outside the droplet)

$$\frac{\partial T{\;}_{g}\left(r,t\right)}{\partial t}=a_{g}^{2}\left(\frac{\partial^{2}T_{g}\left(r,t\right)}{\partial r^{2}}+\frac{2}{r}\frac{\partial T{\;}_{g}\left(r,t\right)}{\partial r}\right)$$ (3)

The quantity *R*(*t*) specifies the motion of the interface with time and is given in eq. (1).

The specification of the boundary conditions completes the statement of the problem. The temperature *T*(*r*, *t*) has the form

$$T_{d}\left(r,t\right)=T_{d0}\;\text{for}\;0\leq r<R\;\text{and}\;t\leq 0$$ (4)

and

$$T_{g}\left(r,t\right)=T_{g0}\;\text{for}\;r>R\;\text{and}\;t\leq 0$$ (5)

and it becomes for *t* > 0

$$T\left(r,t\right)=\{\begin{array}{l} T_{d}\left(r,t\right)\;\text{for}\;0\leq r<R\\ T_{g}\left(r,t\right)\;\text{for}\;r>R. \end{array}$$

The temperature is finite everywhere, namely *T_d_*(0, *t*) is finite and

$$\underset{r\to \infty }{\lim}T_{g}\left(r,t\right)=T_{g0}$$ (6)

The symmetry condition that no heat flux exists at the center of the droplet is

$$\frac{\partial T_{d}\left(0,t\right)}{\partial r}=0$$ (7)

*∂T_d_*(0, *t*)/*∂r* is of course taken to mean the partial derivative of *T_d_*(*r, t*) which respect to r evaluated at *r* = 0. The problem statement for the finite-gradient model is completed by two conditions at the interface *r* = *R* which match the temperature and heat flux

$$T_{d}\left(R^{-},t\right)=T_{g}\left(R^{+},t\right)$$ (8)

$$K_{d}\frac{\partial T_{d}\left(R^{-},t\right)}{\partial r}=K_{g}\frac{\partial T_{g}\left(R^{+},t\right)}{\partial r}+L_{d}d_{d}\frac{dR\left(t\right)}{dt}$$ (9)

where $R^{\pm }\;=\;\underset{\zeta \to 0}{\lim}\left(R\pm \zeta \right)$ and *L_d_* is the latent heat of vaporization per unit mass of the condensed material. From eq (1) we note that *dR*/*dt* goes off to infinity near the end of the droplet lifetime so that the difference between the thermal conductivities times the temperature gradients must become unbounded. More will be said about this point in subsequent sections.

We find it convenient at this point to introduce dimensionless quantities which in this paper will be denoted by Greek letters. They are defined as follows:

1. The time *τ* is chosen to move the singular point of complete droplet evaporation to infinity, $\tau =2\left(a_{d}^{2}/k_{e}\right)$ ln (*r_d_*_0_/*R*(*t*)). As the evaporation rate constant *k_e_* goes to zero *τ* reduces to $\tau_{0}=a_{d}^{2}t/r_{d0}^{2}$.
2. The distance coordinate *η* is chosen to remove the time dependence of the position of the boundary, *η* = *r*/*R*(*t*). Distance is thus measured in instantaneous droplet radii. When *k_e_* = 0, *η* = *η*_0_ = *r*/*r_d_*_0_.
3. The dimensionsless temperature ratios are defined by
  $$\theta_{j}\left(\eta ,\tau \right)=\left(T_{j}\left(r,t\right)-T_{d0}\right)/\left(T_{g0}-T_{d0}\right),\;j=d\;\text{or}\;g.$$
  θ*_d_*(*η*, *τ*) is the fraction of the maximum possible temperature rise. In addition, we define *α = a_d_*/*a*_g_, *β* = *K_g_*/*k_d_*, $\epsilon =k_{e}/a_{d}^{2}$, and *λ* = *L_d_*/[*C_d_*(*T_g_*_0_ − *T_d_*_0_)].

With the above definitions, eqs (2)–(9) become

$$\frac{\partial \theta_{d}\left(\eta ,\tau \right)}{\partial \tau }=\frac{\partial^{2}\theta_{d}\left(\eta ,\tau \right)}{\partial \eta^{2}}+\left(\frac{2}{\eta }-\frac{\epsilon \eta }{2}\right)\frac{\partial \theta_{d}\left(\eta ,\tau \right)}{\partial \eta }\;\left(0\leq \eta <1\right)$$ (10)

$$\alpha^{2}\frac{\partial \theta_{g}\left(\eta ,\tau \right)}{\partial \tau }=\frac{\partial^{2}\theta_{g}\left(\eta ,\tau \right)}{\partial \eta^{2}}+\left(\frac{2}{\eta }-\frac{\alpha^{2}\epsilon \eta }{2}\right)\frac{\partial \theta_{g}\left(\eta ,\tau \right)}{\partial \eta }\;\left(\eta >1\right)$$ (11)

$$\theta_{d}\left(\eta ,0\right)=0$$ (12)

$$\theta_{g}\left(\eta ,0\right)=1$$ (13)

$$\underset{\eta \to \infty }{\lim}\;\theta_{g}\left(\eta ,\tau \right)=1$$ (14)

$$\frac{\partial \theta_{d}\left(0,\tau \right)}{\partial \eta }=0$$ (15)

$$\theta_{d}\left(1^{-},\tau \right)=\theta_{g}\left(1^{+},\tau \right)$$ (16)

$$\frac{\partial \theta_{d}\left(1^{-},\tau \right)}{\partial \eta }=\beta \frac{\partial \theta_{g}\left(1^{+},\tau \right)}{\partial \eta }-\lambda \epsilon /2$$ (17)

Equations (10)–(17) are the complete statement in dimensionless form for the finite-gradient model.

We now consider the zero-gradient model. As was mentioned in section 1, the internal circulation inside the droplet may be sufficient in many cases to maintain a spatially uniform temperature within the droplet. When such conditions prevail, the Fourier heat conduction equation for the droplet, eq (2), and the boundary condition (9) are replaced by a single heat transfer equation. The temperatures in the zero-gradient model are denoted by *T_dz_*(*t*) and *T_gz_*(*r*, *t*) for the droplet and gas respectively. For the case in which the droplet temperature is spatially uniform, *T_d_*(*r*, *t*) = *T_dz_*(*t*) = *T_g_*(*R^+^*, *t*) *= T_gz_*(*R^+^*, *t*) and the heat flux into the droplet,

$$\left(4\pi R^{3}/3\right)d_{d}C_{d}\frac{dT_{dz}\left(t\right)}{dt}/\left(4\pi R^{2}\right)$$ (18)

must equal the heat flux from the gas. Equations (2) and (9) are thus replaced by the following equality

$$\frac{d_{d}C_{d}R}{3}\frac{dT_{dz}\left(t\right)}{dt}=K_{g}\frac{\partial T_{gz}\left(R^{+},t\right)}{\partial r}+L_{d}d_{d}\frac{dR\left(t\right)}{dt}.$$ (19)

Equations (3)–(8) with *T_d_*(*r, t*) and *T_g_*(*r, t*) replaced respectively by *T_dz_*(*t*) and *T_gz_*(*r*, *t*) and eq (19) are the mathematical statement of the zero-gradient model.

In terms of the dimensionless quantities introduced earlier, the working equations in the zero-gradient model are

$$\alpha^{2}\frac{\partial \theta_{gz}\left(\eta ,\tau \right)}{\partial \tau }=\frac{\partial^{2}\theta_{gz}\left(\eta ,\tau \right)}{\partial \eta^{2}}+\left(\frac{2}{\eta }-\frac{\alpha^{2}\epsilon \eta }{2}\right)\frac{\partial \theta_{gz}\left(\eta ,\tau \right)}{\partial \eta }\;\left(\eta >1\right)$$ (20)

$$\theta_{dz}\left(0\right)=0$$ (21)

$$\theta_{gz}\left(\eta ,0\right)=1$$ (22)

$$\underset{\eta \to \infty }{\lim}\;\theta_{gz}\left(\eta ,\tau \right)=1$$ (23)

$$\theta_{dz}\left(\tau \right)=\theta_{gz}\left(1^{+},\tau \right)$$ (24)

$$\frac{1}{3}\frac{d\theta_{dz}\left(\tau \right)}{d\tau }=\beta \frac{\partial \theta_{gz}\left(1^{+},\tau \right)}{\partial \eta }-\frac{\lambda \epsilon }{2}$$ (25)

In the next two sections we solve the equations for the finite-gradient and zero-gradient models by taking their Laplace transforms. The Laplace transform of the reduced temperature θ(*η*, *τ*) is denoted by

$$\Phi \left(\eta ,\sigma \right)=\int_{0}^{\infty }\exp\left(-\sigma \tau \right)\theta \left(\eta ,\tau \right)d\tau$$ (26)

where *σ* is the dimensionless Laplace transform variable. The Bromwich integral

$$\theta \left(\eta ,\tau \right)=\underset{\delta \to \infty }{\lim}\frac{1}{2\pi i}\int_{\gamma -i\delta }^{\gamma +i\delta }\exp\left(\sigma \tau \right)\Phi \left(\eta ,\sigma \right)d\sigma$$ (27)

expresses the temperature in terms of the Laplace transform. The quantity *γ* is chosen sufficiently large so that the integral

$$\int_{0}^{\infty }\exp\left(-\sigma \tau \right)\left|\theta \left(\eta ,\tau \right)\right|d\tau$$

exists.

## 4. Finite-Gradient Model

From eqs (2)–(9), the Laplace transform of the reduced temperature θ(*η*, *τ*) satisfies the following equations and conditions:

$$\left[\frac{\partial^{2}}{\partial \eta^{2}}+\left(\frac{2}{\eta }-\frac{\epsilon \eta }{2}\right)\frac{\partial }{\partial \eta }-\sigma \right]\Phi_{d}\left(\eta ,\sigma \right)=0\;\left(0\leq \eta <1\right)$$ (28)

$$\left[\frac{\partial^{2}}{\partial \eta^{2}}+\left(\frac{2}{\eta }-\frac{\alpha^{2}\epsilon \eta }{2}\right)\frac{\partial }{\partial \eta }-\alpha^{2}\sigma \right]\Phi_{g}\left(\eta ,\sigma \right)+\alpha^{2}=0\;\left(\eta >1\right)$$ (29)

$$\frac{\partial \Phi_{d}\left(0,\sigma \right)}{\partial \eta }=0$$ (30)

$$\underset{\eta \to \infty }{\lim}\Phi_{g}\left(\eta ,\sigma \right)=1$$ (31)

$$\Phi_{d}\left(1^{-},\sigma \right)=\Phi_{g}\left(1^{+},\sigma \right)$$ (32)

$$\frac{\partial \Phi_{d}\left(1^{-},\sigma \right)}{\partial \eta }=\beta \frac{\partial \Phi_{g}\left(1^{+},\sigma \right)}{\partial \eta }-\frac{\lambda \epsilon }{2\sigma }.$$ (33)

To solve the subsidiary differential eqs (28) and (29), we make a change of variable. Letting ζ = *ϵη*^2^/4 gives

$$\left[\zeta \frac{\partial }{\partial \zeta }+\left(\frac{3}{2}-\zeta \right)\frac{\partial }{\partial \zeta }-\frac{\sigma }{\epsilon }\right]\Psi_{d}\left(\zeta ,\sigma \right)=0$$ (34)

$$\left[\alpha^{2}\zeta \frac{\partial^{2}}{\partial {\left(\alpha^{2}\zeta \right)}^{2}}+\left(\frac{3}{2}-\alpha^{2}\zeta \right)\frac{\partial }{\partial \left(\alpha^{2}\zeta \right)}-\frac{\sigma }{\epsilon }\right]\Psi_{g}\left(\alpha^{2}\zeta ,\sigma \right)+\sigma^{-1}=0$$ (35)

where Φ*_d_*(*η*, *σ*) = Ψ*_d_*(*ζ*, *σ*) and Φ*_g_*(*η*, *σ*) = Ψ*_g_*(*α*^2^*ζ*, *σ*).

The operator in square brackets is the confluent hypergeometric differential operator and eqs (34) and (35) are confluent hypergeometric differential equations. We obtain the general solutions to eqs (28) and (29) as

$$\Phi_{d}\left(\eta ,\sigma \right)=AM\left(\sigma /\epsilon ,\;3/2,\;\epsilon \eta^{2}/4\right)+BU\left(\sigma /\epsilon ,\;3/2,\;\epsilon \eta^{2}/4\right)$$ (36)

for the droplet and

$$\Phi_{g}\left(\eta ,\sigma \right)=\sigma^{-1}+CM\left(\sigma /\epsilon ,\;3/2,\;\alpha^{2}\epsilon \eta^{2}/4\right)+DU\left(\sigma /\epsilon ,\;3/2,\;\alpha^{2}\epsilon \eta^{2}/4\right)$$ (37)

for the hot surrounding gas. The quantities *M*(*a, b, z*) and *U*(*a*, *b*, *z*) are two independent solutions to the confluent hypergeometric differential equation and are called confluent hypergeometric functions. Their most useful general properties may be found in Chapter 13 of Ref. [11].

The constants *A, B, C*, and *D* must be determined from the conditions (30)–(33). The condition that no heat flux exists at the center of the droplet requires that *B* = 0 since *U*(*σ*/*ϵ*, 3/2, *ϵη*^2^/4) is not well-behaved at *η* = 0. Similarly, the condition that the reduced gas temperature goes to unity at large distances requires *C* = 0 since *M*(*σ*/*ϵ*, 3/2, *α*^2^*ϵη*^2^/4) is not well-behaved at infinity. The two remaining conditions at the interfacial boundary *η* = 1 constitute two inhomogeneous simultaneous equations for the remaining constants *A* and *D.* Solving these, we obtain the Laplace transformed solutions for the reduced temperatures:

$$\Phi_{d}\left(\eta ,\sigma \right)=\frac{3\left[\alpha^{2}\beta \sigma U\left(\frac{\sigma }{\epsilon }+1,\frac{5}{2},\frac{\alpha^{2}\epsilon }{4}\right)-\lambda \epsilon U\left(\frac{\sigma }{\epsilon },\frac{3}{2},\frac{\alpha^{2}\epsilon }{4}\right)\right]M\left(\frac{\sigma }{\epsilon },\frac{3}{2},\frac{\epsilon \eta^{2}}{4}\right)}{\sigma^{2}H\left(\sigma /\epsilon \right)}$$ (38)

for the droplet, and

$$\Phi_{g}\left(\eta ,\sigma \right)=\sigma^{-1}-\frac{\left[2\sigma M\left(\frac{\sigma }{\epsilon }+1,\frac{5}{2},\frac{\epsilon }{4}\right)+3\lambda \epsilon M\left(\frac{\sigma }{\epsilon },\frac{3}{2},\frac{\epsilon }{4}\right)\right]U\left(\frac{\sigma }{\epsilon },\frac{3}{2},\frac{\alpha^{2}\epsilon \eta^{2}}{4}\right)}{\sigma^{2}H\left(\sigma /\epsilon \right)}$$ (39)

for the gas where

$$H\left(\sigma /\epsilon \right)=2M\left(\frac{\sigma }{\epsilon }+1,\frac{5}{2},\frac{\epsilon }{4}\right)U\left(\frac{\sigma }{\epsilon },\frac{3}{2},\frac{\alpha^{2}\epsilon }{4}\right)+3\alpha^{2}\beta M\left(\frac{\sigma }{\epsilon },\frac{3}{2},\frac{\epsilon }{4}\right)U\left(\frac{\sigma }{\epsilon }+1,\frac{5}{2},\frac{\alpha^{2}\epsilon }{4}\right).$$ (40)

The differential relations of *M* and *U* have been used in deriving these equations. These expressions have been derived in a form suitable for going to the limit of zero evaporation rate constant, *ϵ* = 0. The correct limiting cases may be found in Ref. [6] and the details of the limiting process in appendix A.

The reduced temperature distributions of the droplet and surrounding gas are obtained by evaluating the Bromwich integrals of eq (8):

$$\theta \left(\eta ,\tau \right)=\underset{\delta \to \infty }{\lim}\frac{1}{2\pi i}\int_{\gamma +i\delta }^{\gamma +i\delta }{\;}_{\gamma -i\delta \exp}\left(\sigma \tau \right)\Phi \left(\eta ,\sigma \right)d\sigma .$$ (41)

To evaluate the Bromwich integrals it is first necessary to investigate the nature of the integrand exp (*στ*)Φ(*η*, *σ*). No distinction will be made in this discussion between the droplet and the gas as the integrands in both cases exhibit the same general properties. First of all, exp (*στ*)Φ(*η*, *σ*) is a single-valued, analytic function of *σ* except at its singularities. It has a simple second order pole at *σ* = 0 and an infinity of real, negative simple first order poles at the zeroes of *H*(*σ*/*ϵ*) given in eq (40). To show that *H*(*σ*/*ϵ*) possesses no complex, imaginary or positive zeros is straight-foreward. General methods for locating such zeros may be found, for example, in Carslaw and Jaeger [12] and Ince [13].

We now evaluate the Bromwich integral. We complete the contour of eq (41) by a large semicircular arc in the left-hand complex plane and take *γ* to be an arbitrarily small positive number. The final reduced temperature distributions are then obtained by applying the Cauchy Residue Theorem and letting *δ* and the radius of the large semicircular arc tend to infinity. The integral along the large semicircular arc vanishes and we find

$$\theta_{d}\left(\eta ,\tau \right)=\frac{3}{H\left(0\right)}\left[{\frac{\lambda }{H\left(0\right)}\frac{\partial H\left(\rho \right)}{\partial \rho }|}_{\rho =0}{-\lambda \frac{\partial U\left(\rho ,\frac{3}{2},\frac{\alpha^{2}\epsilon }{4}\right)}{\partial \rho }|}_{\rho =0}{-\lambda \frac{\partial M\left(\rho ,\frac{3}{2},\frac{\epsilon \eta^{2}}{4}\right)}{\partial \rho }|}_{\rho =0}+\alpha^{2}\beta U\left(1,\frac{5}{2},\frac{\alpha^{2}\epsilon }{4}\right)-\epsilon \lambda \tau \right]+3\sum_{n=1}^{\infty }\frac{\exp\left(\epsilon \rho_{n}\tau \right)\left[\rho_{n}\alpha^{2}\beta U\left(\rho_{n}+1,\frac{5}{2},\frac{\alpha^{2}\epsilon }{4}\right)+\lambda U\left(\rho_{n},\frac{3}{2},\frac{\alpha^{2}\epsilon }{4}\right)\right]M\left(\rho_{n},\frac{3}{2},\epsilon \eta^{2}/4\right)}{{\rho^{2}{\;}_{n}\partial H\left(\rho \right)/\partial \rho |}_{\rho =\rho_{n}}}.$$ (42)

and

$$\theta_{g}\left(\eta ,\tau \right)=1+\frac{1}{H\left(0\right)}\left[{\frac{3\lambda }{H\left(0\right)}\frac{\partial H\left(\rho \right)}{\partial \rho }|}_{\rho =0}{-3\lambda \frac{\partial M\left(\rho ,\frac{3}{2},\frac{\epsilon }{4}\right)}{\partial \rho }|}_{\rho =0}{-3\lambda \frac{\partial U\left(\rho ,\frac{3}{2},\frac{\alpha^{2}\epsilon \eta^{2}}{4}\right)}{\partial \rho }|}_{\rho =0}-2M\left(1,\frac{5}{2},\frac{\epsilon }{4}\right)-3\epsilon \lambda \tau \right]-\sum_{n=1}^{\infty }\frac{\exp\left(\epsilon \rho_{n}\tau \right)\left[2\rho_{n}M\left(\rho_{n}+1,\frac{5}{2},\frac{\epsilon }{4}\right)+3\lambda M\left(\rho_{n},\frac{3}{2},\frac{\epsilon }{4}\right)\right]U\left(\rho_{n},\frac{3}{2},\alpha^{2}\epsilon \eta^{2}/4\right)}{{\rho^{2}{\;}_{n}\partial H\left(\rho \right)/\partial \rho |}_{\rho =\rho_{n}}}.$$ (43)

where *H*(*ρ_n_*) = 0, *n* = 1, 2, 3 ⋯; i.e., the *ρ_n_* are the zeros of *H*(*ρ*).

We pause to make several comments about the expressions obtained for θ*_d_*(*η*, *τ*) and θ*_g_*(*η*, *τ*). Both contain a term proportional to the reduced time *τ* which is a consequence of the second order pole at *σ* = 0. As mentioned after eq (9), the choice of linear surface regression rate implies that the difference between the thermal conductivities times the temperature gradients evaluated at the interface goes to infinity at large times. By large times of course, we mean times close to the end of the droplet’s lifetime. These are times for which the linear surface regression rate of eq (1) is no longer an accurate representation of the true physical situation. For A, *ϵ*, and *τ* of the orders of magnitude of interest here, the linear term in *τ* may be considered an artifact of the model and makes only a very small contribution to the reduced temperatures. We remark also that eqs (42) and (43) are not suitable for going directly to the limit *ϵ* = 0. Further discussion of this point may be found in appendix A.

In section 6 are presented plots of θ*_d_*(1, *τ*) versus *τ* for typical values of the dimensionless quantities *λ* and *ϵ.* In the next section we derive expressions for the reduced temperatures for the zero-gradient model.

## 5. Zero-Gradient Model

The analysis for the zero-gradient model is mathematically very similar to that for the finite-gradient model and will not be quite as detailed. Equations (20)–(25) are the working equations for the zero-gradient model. The Laplace transformed system of equations is

$$\left[\frac{\partial^{2}}{\partial \eta^{2}}+\left(\frac{2}{\eta }-\frac{\alpha^{2}\epsilon \eta }{2}\right)\frac{\partial }{\partial \eta }-\alpha^{2}\sigma \right]\Phi_{gz}\left(\eta ,\sigma \right)+\alpha^{2}=0$$ (44)

$$\underset{\eta \to \infty }{\lim}\Phi_{gz}\left(\eta ,\sigma \right)=1$$ (45)

$$\Phi_{dz}\left(\sigma \right)=\Phi_{gz}\left(1^{+},\sigma \right)$$ (46)

$$\frac{\sigma }{3}\Phi_{\partial z}\left(\sigma \right)=\beta \frac{\partial \Phi_{gz}\left(1^{+},\sigma \right)}{\partial \eta }-\frac{\lambda \epsilon }{2\sigma }$$ (47)

The general solution to the differential eq (44) which satisfies the condition (45) is

$$\Phi_{gz}\left(\eta ,\sigma \right)=\sigma^{-1}+AU\left(\sigma /\epsilon ,3/2,\alpha^{2}\epsilon \eta^{2}/4\right)$$ (48)

Combining eqs (46) and (47) to eliminate Φ*_dz_*(*σ*) yields the relation

$$\frac{\sigma }{3}\Phi_{gz}\left(1^{+},\sigma \right)=\beta \frac{\partial \Phi_{gz}\left(1^{+},\sigma \right)}{\partial \eta }-\frac{\lambda \epsilon }{2\sigma }$$ (49)

which is then used to determine the single unknown constant *A* of eq (48). Doing this yields the Laplace transformed reduced temperature distributions

$$\Phi_{dz}\left(\sigma \right)=\Phi_{gz}\left(1^{+},\sigma \right)$$ (50)

for the droplet, and

$$\Phi_{gz}\left(\eta ,\sigma \right)=\sigma^{-1}-\frac{\left(2\sigma +3\lambda \epsilon \right)U\left(\sigma /\epsilon ,3/2,\alpha^{2}\epsilon \eta^{2}/4\right)}{\sigma^{2}I\left(\sigma /\epsilon \right)}$$ (51)

for the gas where

$$I\left(\sigma /\epsilon \right)=2U\left(\sigma /\epsilon ,3/2,\alpha^{2}\epsilon /4\right)+3\beta \alpha^{2}U\left(1+\sigma /\epsilon ,5/2,\alpha^{2}\epsilon /4\right)$$ (52)

These expressions have been derived in a form suitable for going to the limit *ϵ* = 0, which represents the nonevaporative problem solved in Ref. [6]. That they in fact reduce to the correct limit is shown in appendix A.

All that remains to be done is to evaluate the Bromwich integral of eq (8) with Φ(*η*, *σ*) = Φ*_gz_*(*η*, *σ*)

$$\theta_{gz}\left(\eta ,\tau \right)=\underset{\delta \to \infty }{\lim}\frac{1}{2\pi i}\int_{\gamma -i\delta }^{\gamma +i\delta }\exp\left(\sigma \tau \right)\Phi_{gz}\left(\eta ,\sigma \right)d\sigma .$$ (53)

The same general comments made about the corresponding integrands in the finite-gradient model are applicable here also. We complete the contour by a large semicircular arc in the left-hand complex plane, apply the Cauchy Residue Theorem and let *δ* and the radius of the semicircular arc tend to infinity. Following this sequence of steps were find

$$\theta_{dz}\left(\tau \right)=\theta_{gz}\left(1^{+},\tau \right)$$ (54)

and

$$\theta_{gz}\left(\eta ,\tau \right)=1+\frac{1}{I\left(0\right)}\left[{\frac{3\lambda }{I\left(0\right)}\frac{\partial I\left(\rho \right)}{\partial \rho }|}_{\rho =0}{-2-3\lambda \frac{\partial U\left(\rho ,3/2,\alpha^{2}\epsilon \eta^{2}/4\right)}{\partial \rho }|}_{\rho =0}-3\epsilon \lambda \tau \right]-\sum_{\eta =1}^{\infty }\frac{\exp\left(\epsilon \rho_{n}\tau \right)\left(2\rho_{n}+3\lambda \right)U\left(\rho_{n},3/2,\alpha^{2}\epsilon \eta^{2}/4\right)}{{\rho^{2}{\;}_{n}\partial I\left(\rho \right)/\partial \rho |}_{\rho =\rho_{n}}}$$ (55)

where *I*(*ρ_n_*) = 0, *n* = 1, 2, 3, ⋯; i.e., the *ρ_n_* are the negative real zeros of *I*(*ρ*). Note that the linear term in *τ* also appears in the zero-gradient model solutions.

It is possible to obtain some very simple and accurate approximations to eqs (54) and (55). The thermal diffusivity of the droplet is several orders of magnitude smaller than that of the surrounding gas. This suggests that before applying the Laplace inversion formula we expand the subsidiary solution Φ*_gz_* (*η*, *σ*) into a power series in *α* = *a_d_*/*a_g_*. We obtain only the simplest approximation by letting *α* go to zero in eq (51):

$$\underset{\alpha \to 0}{\lim}\Phi_{gz}\left(\eta ,\sigma \right)\equiv \Phi_{g0}\left(\eta ,\sigma \right)=\sigma^{-1}-\frac{2\sigma +3\epsilon \lambda }{2\eta \sigma \left(\sigma +3\beta \right)}$$ (56)

The inverse Laplace transform of this remarkably simple expression yields the following approximations for the reduced temperatures in the zero-gradient model.

$$\theta_{d0}\left(\tau \right)=\left(1-\frac{\lambda \epsilon }{2\beta }\right)\left(1-\exp\left(-3\beta \tau \right)\right)$$ (57)

for the droplet, and

$$\theta_{g0}\left(\eta ,\tau \right)=1-\eta^{-1}\exp\left(-3\beta \tau \right)-\left(\lambda \epsilon /2\beta \eta \right)\left(1-\exp\left(-3\beta \tau \right)\right)$$ (58)

for the surrounding gas. Note that the term linear in *τ* has disappeared to the order of approximation used. However, if Φ*_gz_*(*η*, *σ*) is expanded out to first order in *α*, then a relatively simple infinite series and the linear term in *τ* show up in the approximation of θ*_gz_*(*η*, *τ*). Unfortunately, the expressions obtained by letting *α* go to zero in the finite-gradient model are much the same as the exact solutions.

In the next section, we present plots of θ*_dz_*(*τ*) versus *τ* for typical values of *λ* and *ϵ.* In addition, the times predicted by θ*_d_*(*η*, *τ*), θ*_dz_*(*η*, *τ*) and θ*_d_*_0_(*τ*) for the droplet to reach a given temperature are compared and discussed.

## 6. Numerical Examples and Conclusions

In this section, we give some illustrative numerical examples for the predictions made by finite-gradient and zero-gradient models. The input data for the calculations are the four dimensionless quantities *α*, *β*, *λ* and *ϵ*. We take *α* = 0.057, *β* = 0.186 and *λ* = 0.01, 0.055, 0.10 and *ϵ* = 0.10, 0.55, 1.0, independently, so that there are nine cases. These values correspond roughly to those of medium weight fuel oil in hot air at atmospheric pressure. Typical values of the physical parameters K, d, and C which yield the chosen values of *α* and *β* are given in table 1. In table 2, we give the values of L_d_ corresponding to *λ* = 0.01, 0.055, and 0.10 and k_e_ corresponding to *ϵ* = 0.10, 0.55, and 1.0. The temperature difference *T_g_*_0_ − *T_d_*_0_ is taken to be 1666.67 K (corresponding to *T_d_*_0_ = 300 K and *T_g_*_0_ = 1966.67 K) and $a_{d}^{2}$ and *C_d_* are assigned the values given them in table 1.

**Figure 1:**
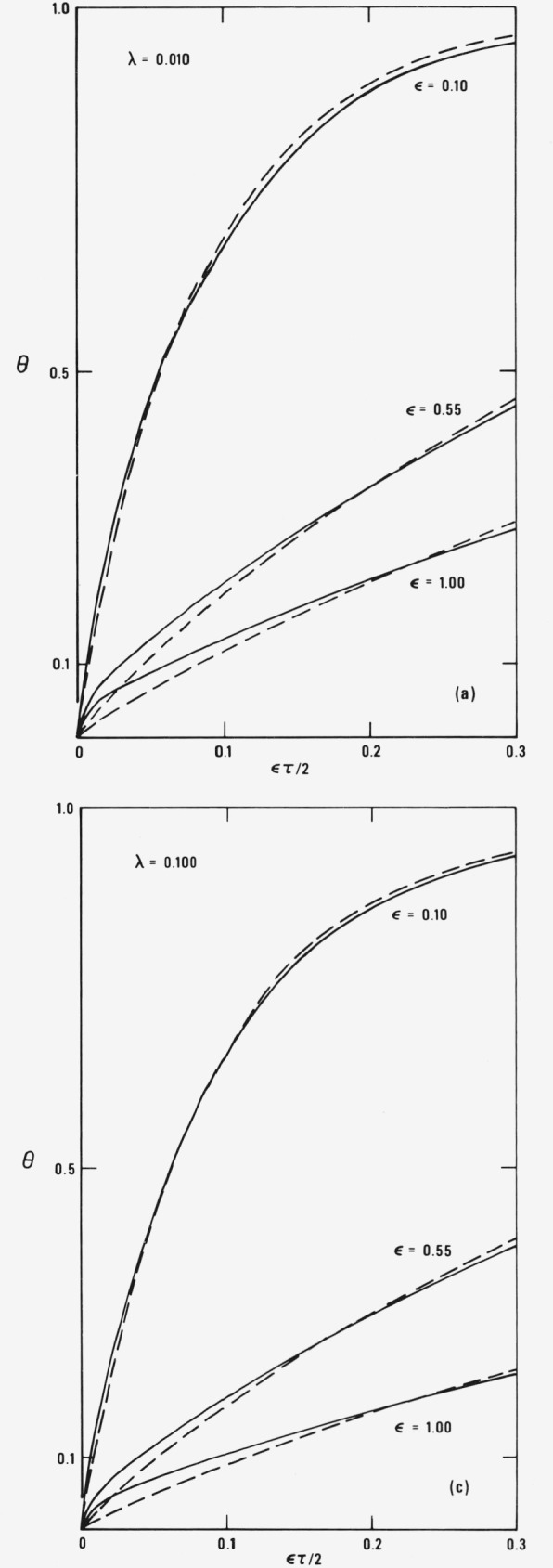
The reduced, temperature *θ*_d_(1^−^, τ) (solid lines) and *θ*_dz_(τ) (dashed lines) plotted as a function of ϵτ/2 with α = 0.057, β = 0.186 and ϵ = 0.10, 0.55, and 1.0 for λ equal to (a) 0.01, (b) 0.055, and (c) 0.10. The initial droplet radius, r_d0_, is 50 μm.

Figures 1a, 1b, and 1c compare the numerical predictions of the finite-gradient and zero-gradient models for oil droplets in air for *λ* equal to 0.01, 0.055, and 0.10, respectively. θ*_d_*(1^−^, *τ*) and θ*_dz_*(*τ*), the reduced surface temperatures, are computed from eqs (42) and (54). It should be noted that due to the presence of the linear term in *τ*, neither θ*_d_*(1^−^, *τ*) nor θ*_dz_*(*τ*) reach steady state during the droplet lifetime. Even if that term were absent, θ*_d_*(*η*, *τ*) would never become uniform throughout the droplet. We must stress at this point that the apparent large difference between the temperature profiles for fixed *λ* but different *ϵ* is deceiving. Although shown on the same graph, the curves are plotted versus *ϵτ*/2 which is a function of *ϵ*, i.e., each curve has its own distinct time scale. The results have been shown in this fashion for clarity only. If they were all plotted on the same scale, real time *t* for example, the entire group of curves would lie very close to one another. As can be seen from the figures, θ*_d_*/(1^−^, *τ*) and θ*_dz_*(*τ*) are quite similar. Their difference is greatest when *ϵτ*/2 is small. This is to be expected. The thermal gradients inside the droplet are largest near the beginning of its lifetime and for such times the approximations of the zero-gradient model are more suspect. Although not plotted, the simple approximation θ*_d_*_0_(*τ*) to θ*_dz_*(*τ*) obtained in the last section, is an extremely good one as long as *ϵτ*/2 is neither too large nor too small. The difference between θ*_dz_*(*τ*) and θ*_d_*_0_(*τ*) is less than about 15 percent in the worst case (*λ* = 0.1 and *ϵ* = 1.0) and much smaller in the other cases for the *τ*’s we have considered. Due to its simple analytic form, accuracy, and ease of mathematical computation, we suspect that θ*_d_*_0_(*τ*) is potentially a very useful expression for analyses of the initial preheat period of droplets in hot atmospheres.

**Figure 2:**
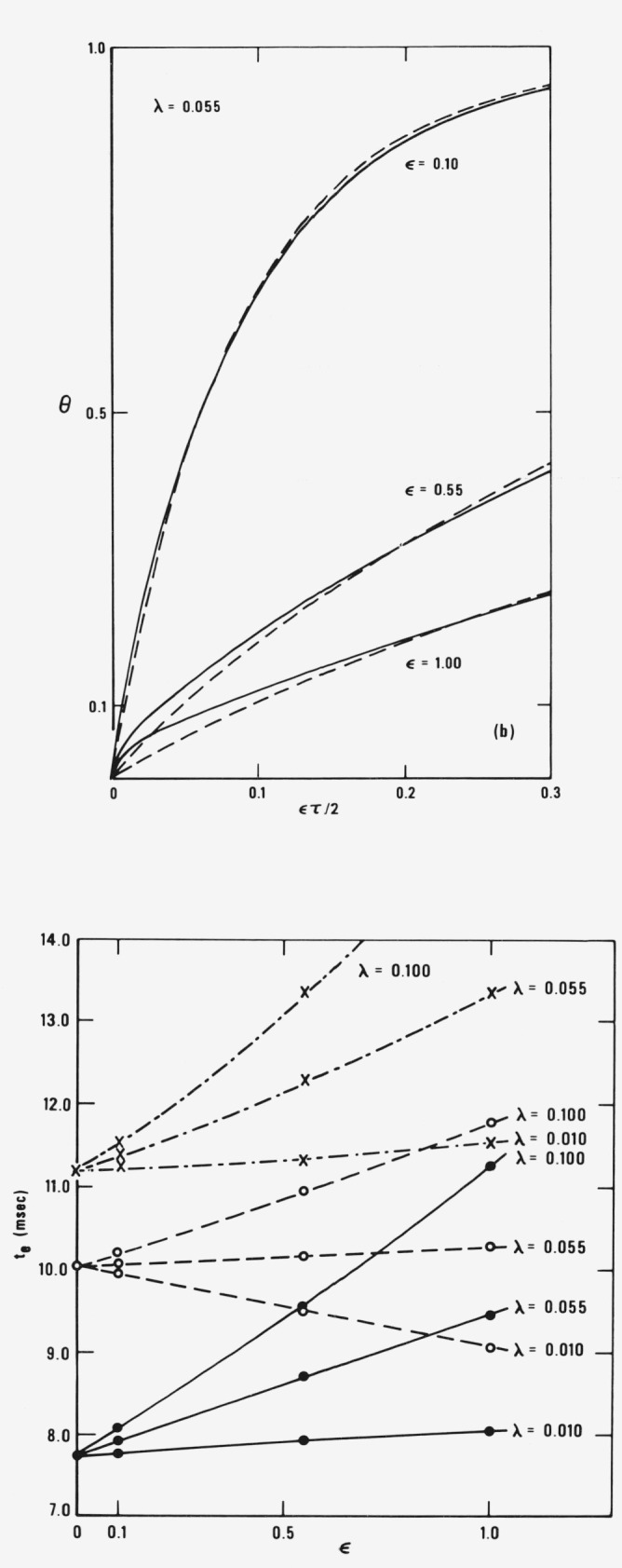
The times t_e_ required for a droplet to reach obtained from *θ*_d_(1^−^, τ) (solid lines and closed circles), *θ*_dz_(τ) (dashed lines and crosses) plotted as a function of ϵ for various values of λ. *The physical properties used are those of* table 1 and the initial droplet radius, r*_∂_*_0_, *is 50 μm.* The temperature *T_s_* corresponds to *θ*(*t_e_*) = 0.15.

A quantity of interest in the design of combustion chambers is the time required for fuel droplet to reach a given temperature *T_s_.* Let us denote this time by *t_e_* and consider a fuel droplet with the same physical parameters given in table 1 and an initial radius *r_d_*_0_ = 50 *μ*m. For illustrative purposes, let us assume that *T*_SL_ = 550 K, the initial droplet temperature *T_d_*_0_ = 300 K, and the initial gas temperature *T_g_*_0_ = 1966.67 K. The droplet reading *T_s_* corresponds to θ*_S_* = 0.15. Using the data displayed graphically as figs, 1a, 1b, and 1c, we compute the times required for θ to increase from zero to 0.15. The results are shown in figure 2 as a plot of *t_e_* versus *ϵ*. It can be seen that the times, *t_e_*, predicted by the finite-gradient and zero-gradient models are within about 20 percent of each other. For comparison, the $t_{e}^{’}s$ predicted by θ*_d_*_0_(*τ*) are also presented and are seen to be within 10–20 percent of those predicted by θ*_dz_*(*τ*) as long as *ϵ* ⪝ 0.55. Figure 2 also provides an extremely powerful cross check on the analytic and numerical results of this paper and those of Ref. [9]. By solving a totally different problem, Bennett and Kayser [9] obtained the times *t_e_* for the case of *ϵ* = 0. These are the points on the *t*-axis of figure 2. By extrapolating the results obtained here for nonzero *ϵ* to *ϵ* equal to zero, it can be seen that the results are in agreement and this indicates that this work and that of Ref. [9] are internally consistent.

**Figure 3:**
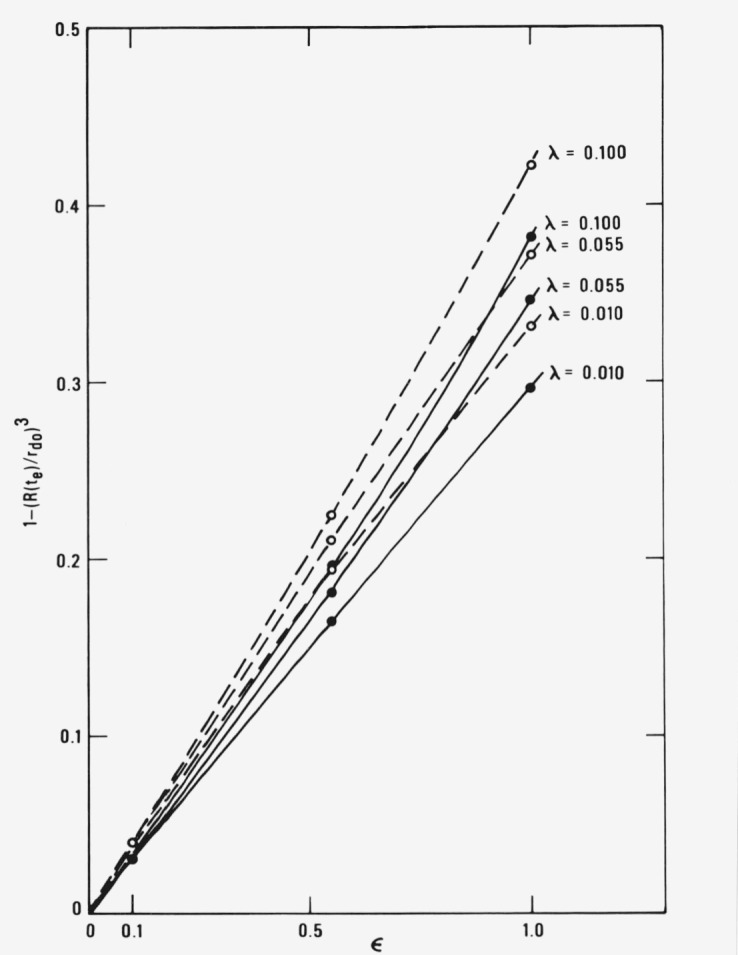
Volume fraction of a droplet that has evaporated in the time t_e_ plotted as a function of ϵ for various values of λ. The closed circles and solid lines are for the finite-gradient model (θ*_d_*(1^−^, *τ*)) and the open circles and dashed lines are for the zero-gradient model (θ*_dz_*(*τ*)). The physical properties used are those in table 1 and the initial droplet radius, *r_d_*_0_, is 50 *μ*m. The times *t_e_* are those of figure 2.

Figure 3 contains a plot of the volume fraction of the droplet that has evaporated away by the time *t* = *t_e_* versus *ϵ*. For *ϵ* = 0.1, an evaporation rate constant fairly characteristic of the initial preheat stage, the volume fraction evaporated is less than 5 percent. If *ϵ* is fairly small, this perhaps justifies neglecting evaporation and surface regression rate in analyzing the initial droplet preheat stage. A fairly decent approximate procedure for predicting the times *t_e_* and the droplet size at *t = t_e_* is to neglect evaporation, compute the time required for the droplet to reach a given temperature, and then to use that time and a relation like eq (1) to calculate the volume fraction evaporated.

## Figures and Tables

**Table 1:** Typical values of the thermal conductivity *K*, density *d*, specific heat *C* at constant volume, and thermal diffusivity *a^2^* which yield α = *a_d_/a_g_* = 0.057 and β = *Kg/K_d_* = 0.186

Property	Droplet	Gas
		
*K*(J/cm s °C)	1.45 × 10^−3^	2.69 × 10^−4^
*d*(g/cm^3^)	0.90	1.3 × 10^−3^
*C*(J/g °C)	2.39	1.0
*a*^2^(cm^2^/s)	6.5 × 10^−4^	0.20

**Table 2:** The values of the droplet latent heat of vaporization per gram, *L_d_*, and the evaporation rate constant, *k_e_*, corresponding to the values of the dimensionless parameters λ = *L_d_*/{*C_d_*(*T_g0_* − *T_d0_*)} and ϵ = *k_e_/a_d_*^2^ used. The temperature difference T_g0_ − T_d0_ is taken to be 1666.67 K and $a_{d}^{2}$ and *C_d_* are assigned the values given in table 1.

*λ*	*L_d_*(J/g)	*ϵ*	*k_e_*(cm^2^/s)
			
0.01	39.83	0.1	6.5 × 10^−5^
0.055	219.08	0.55	3.575 × 10^−4^
0.10	398.33	1.0	6.5 × 10^−4^

## Footnotes

1 Figures in brackets indicate the literature references at the end of this paper.

## 7. Appendix A

In this appendix, we show that intermediate results such as those of eqs (38) and (51) reduce to the correct limits as *ϵ*, the reduced evaporation rate constant, goes to zero. We also make some general remarks concerning this limiting process.

The results of the *ϵ* = 0 problem corresponding to eqs (38) and (51) will be denoted by a superscript ′0′ and are [6]:

$$\Phi_{d}^{0}\left(\eta_{0},\sigma \right)=\frac{\beta \left(1+\alpha \sigma^{1/2}\right)\sin h\;\left(\sigma^{1/2}\eta_{0}\right)}{\sigma \eta_{0}\left[\left\{\beta \left(1+\alpha \sigma^{1/2}\right)-1\right\}\sin h\;\sigma^{1/2}+\sigma^{1/2}\cos h\;\sigma^{1/2}\right]}$$ (A1)

for the droplet in the finite-gradient model and

$$\Phi_{gz}^{0}\left(\eta_{0},\sigma \right)=\sigma^{-1}-\exp\left[-\alpha \sigma^{1/2}\left(\eta_{0}-1\right)\right]/\left[\eta_{0}\left(\sigma +3\beta \alpha \sigma^{1/2}+3\beta \right)\right]$$ (A2)

for the gas in the zero-gradient model. The quantities *η*_0_ and

*τ*_0_ are defined in section 3. We use the relations [11]

dM(a,b,z)/dz=aM(a+1,b+1,z)/b (A3)

dU(a,b,z)/dz−aU(a+1,b+1,z) (A4)

to rewrite eqs (38) and (51) as

$$\Phi_{d}\left(\eta ,\sigma \right)=\frac{-\left[{\beta \frac{d}{d\eta }U\left(\frac{\sigma }{\epsilon },\frac{3}{2},\frac{\alpha^{2}\epsilon \eta^{2}}{4}\right)|}_{\eta =1}-\frac{1}{4}\epsilon \lambda U\left(\frac{\sigma }{\epsilon },\frac{3}{2},\frac{\alpha^{2}\epsilon }{4}\right)\right]M\left(\frac{\sigma }{\epsilon },\frac{3}{2},\frac{\epsilon \eta^{2}}{4}\right)}{\sigma \left[{\frac{d}{d\eta }M\left(\frac{\sigma }{\epsilon },\frac{3}{2},\frac{\epsilon \eta^{2}}{4}\right)|}_{\eta =1}{U\left(\frac{\sigma }{\epsilon },\frac{3}{2},\frac{\alpha^{2}\epsilon }{4}\right)-\beta M\left(\frac{\sigma }{\epsilon },\frac{3}{2},\frac{\epsilon }{4}\right)\frac{d}{d\eta }U\left(\frac{\sigma }{\epsilon },\frac{3}{2},\frac{\alpha^{2}\epsilon \eta^{2}}{4}\right)|}_{\eta =1}\right]}$$ (A5)

$$\Phi_{gz}\left(\eta ,\sigma \right)=\sigma^{-1}+\frac{\left(2\sigma +3\epsilon \lambda \right)U\left(\sigma /\epsilon ,3/2,\alpha^{2}\epsilon \eta^{2}/4\right)}{2\sigma \left[{3\beta \left(d/d\eta \right)U\left(\sigma /\epsilon ,3/2,\alpha^{2}\epsilon \eta^{2}4\right)|}_{n=1}-\sigma U\left(\sigma /\epsilon ,3/2,\alpha^{2}\epsilon /4\right)\right]}$$ (A6)

Again from Ref. [11] we obtain

$$\underset{a\to \infty }{\lim}\left[M\left(a,b,z/a\right)/\Gamma \left(b\right)\right]=z^{1/2-1/2b}I_{b-1}\left(2z^{1/2}\right)$$ (A7)

$$\underset{a\to \infty }{\lim}\left[\Gamma \left(1+a-b\right)U\left(a,b,z/a\right)\right]=2z^{1/2-1/2}K_{b-1}\left(2z^{1/2}\right)$$ (A8)

where *I* and *K* are modified Bessel functions. Using these two limiting expressions, we find

$$\underset{\epsilon \to 0}{\lim}M\left(\sigma /\epsilon ,3/2,\epsilon \eta^{2}/4\right)/\Gamma \left(3/2\right)]=\frac{2\;\sin h\;\left(\sigma^{1/2}\eta_{0}\right)}{\sqrt{\pi }\eta_{0}\sigma^{1/2}}$$ (A9)

$$\underset{\epsilon \to 0}{\lim}\left[\Gamma \left(\frac{\sigma }{\epsilon }-\frac{1}{2}\right)U\left(\sigma /\epsilon ,3/2,\alpha^{2}\epsilon \eta^{2}/4\right)\right]=\frac{2\sqrt{\pi }\exp\left(-\alpha \eta_{0}\sigma^{1/2}\right)}{\alpha \eta_{0}\sigma^{1/2}}.$$ (A10)

Multiplying the numerator and denominator of eq (A5) by $\Gamma \left(\frac{\sigma }{\epsilon }-\frac{1}{2}\right)/\Gamma \left(\frac{3}{2}\right)$, substituting the results of eqs. (A9) and (A10), replacing *d*/*dη* by *d*/*dη_0_*, and rearranging the resulting expression yields eq (A1); that is

$$\underset{\epsilon \to 0}{\lim}\Phi_{d}\left(\eta ,\sigma \right)=\Phi_{d}^{0}\left(\eta_{0},\sigma \right).$$ (A11)

Multiplying numerator and denominator of eq (A6) by $\Gamma \left(\frac{\sigma }{\epsilon }-\frac{1}{2}\right)$ and then performing the same sequence of operations as for the finite-gradient model leads to eq (A2):

$$\underset{\epsilon \to 0}{\lim}\Phi_{gz}\left(\eta ,\sigma \right)=\Phi_{gz}^{0}\left(\eta_{0},\sigma \right).$$ (A12)

The same general procedure may easily be used to show that other intermediate results also tend to the correct limit as *ϵ* goes to zero.

It is the intermediate results which may be checked and not the final results obtained by evaluating the Bromwich integrals. It is not permissible, as may be verified by trying it, to take the expressions θ*_d_*(*η*, *τ*) and θ*_gz_*(*η*, *τ*) of eqs (42) and (55) to the limit of *ϵ* being zero to obtain $\theta_{d}^{0}\left(\eta_{0},\tau_{0}\right)$ and $\theta_{gz}^{0}\left(\eta_{0},\tau_{0}\right)$. This limiting process is improper for the following reason. Inspection of eqs (A1), (A2), (A5) and (A6) shows the first two to be double-valued functions of *σ* with a branch points at *σ* = 0 and the last two to be single-valued analytic functions of σ. As one proceeds to the limit of zero *ϵ*, an infinity of simple first order poles coalesces into a branch point at the origin, *σ* = 0. The evaluation of the Bromwich integrals leading to θ*_d_*(*η*, *τ*) and θ*_gz_*(*η*, *τ*) is thus only valid for nonzero *ϵ*. The expressions representing θ*_d_*(*η*, *τ*) and θ*_gz_*(*η*, *τ*) only do so for nonzero 6 and this explains why it is impermissible, in fact impossible, to obtain $\theta_{d0}^{0}\left(\eta_{0},\tau_{0}\right)$ and $\theta_{gz}^{0}\left(\eta_{0},\tau_{0}\right)$ from them by any limiting process.
